# Machine Learning Techniques for Hypoglycemia Prediction: Trends and Challenges

**DOI:** 10.3390/s21020546

**Published:** 2021-01-14

**Authors:** Omer Mujahid, Ivan Contreras, Josep Vehi

**Affiliations:** 1 Model Identification and Control Laboratory, Institut d’Informatica i Applicacions, Universitat de Girona, 17003 Girona, Spain; omer.mujahid@udg.edu (O.M.); ivancontreras@udg.edu (I.C.); 2 Centro de Investigación Biomédica en Red de Diabetes y Enfermedades Metabólicas Asociadas (CIBERDEM), 17003 Girona, Spain

**Keywords:** hypoglycemia, machine learning, prediction, detection, artificial intelligence, decision support system (DSS)

## Abstract

(1) Background: the use of machine learning techniques for the purpose of anticipating hypoglycemia has increased considerably in the past few years. Hypoglycemia is the drop in blood glucose below critical levels in diabetic patients. This may cause loss of cognitive ability, seizures, and in extreme cases, death. In almost half of all the severe cases, hypoglycemia arrives unannounced and is essentially asymptomatic. The inability of a diabetic patient to anticipate and intervene the occurrence of a hypoglycemic event often results in crisis. Hence, the prediction of hypoglycemia is a vital step in improving the life quality of a diabetic patient. The objective of this paper is to review work performed in the domain of hypoglycemia prediction by using machine learning and also to explore the latest trends and challenges that the researchers face in this area; (2) Methods: literature obtained from PubMed and Google Scholar was reviewed. Manuscripts from the last five years were searched for this purpose. A total of 903 papers were initially selected of which 57 papers were eventually shortlisted for detailed review; (3) Results: a thorough dissection of the shortlisted manuscripts provided an interesting split between the works based on two categories: hypoglycemia prediction and hypoglycemia detection. The entire review was carried out keeping this categorical distinction in perspective while providing a thorough overview of the machine learning approaches used to anticipate hypoglycemia, the type of training data, and the prediction horizon.

## 1. Introduction

Hypoglycemia is the drop in blood glucose (BG) below critical levels [1]. The BG level at which hypoglycemia occurs, however, has long been a topic of much debate in medical circles [2]. The most accepted definition is that when the BG level drops below 70 mg/dL or 3.9 mmol/L, hypoglycemia is diagnosed [3]. It is one of the most lethal conditions that may arise most commonly in type 1 diabetics (T1D) followed by type 2 diabetics (T2D). Hypoglycemia may lead to loss of consciousness, confusion, seizures, and in extreme cases, death [4]. The symptoms of hypoglycemia, however, may vary for different individuals based on several factors. For a symptom to be associated with hypoglycemia, it is important that it satisfies Whipple’s triad [5]. This essentially means that the symptom is consistent with hypoglycemia, the blood glucose level is below the normal range, and the symptom is relieved when the plasma glucose level is increased to normal or above. The symptoms of hypoglycemia are eventually connected to neuronal glucose deprivation, which in layman terms means glucose deprivation of the human nervous system and brain [6]. These symptoms could then be categorized into neurogenic symptoms caused by glucose deprivation of the autonomic nervous system and the neuroglycopenic symptoms caused by the glucose deprivation of the central nervous system. Based on the level of BG, the symptoms of hypoglycemia can be categorized as mild, moderate, and extreme. It is in the extreme form that hypoglycemia is most lethal. In most cases, however, hypoglycemia does not have any symptoms at all and occurs silently. The silent arrival of hypoglycemia is one of the causes of distress for its sufferers. Along with the physical discomforts that hypoglycemia brings, the mental torments are a major reason for the diabetics to despise its existence. The insecurity and fear of hypoglycemia causes the life quality of diabetics to degrade immensely [7]. The main cause of hypoglycemia is the reduction in blood glucose levels because of an overdose of insulin or a low intake of food/carbohydrates [8]. The reduction in BG that is caused by insulin or any other form of drugs is known as iatrogenic hypoglycemia [9]. Hypoglycemia may occur because of multiple other reasons, i.e., kidney failure, liver complications, hyperthyroidism, starvation, and the consumption of certain drugs including alcohol. Hypoglycemia may be classified into multiple groups based on factors, i.e., the agent of cause, the time of the day it occurs, the age of the individual, the severity of the glycemic event, and the connection to another condition in the body [10].

Based on the time of occurrence, hypoglycemia is commonly characterized into daytime hypoglycemia, postprandial hypoglycemia [11], and nocturnal hypoglycemia [10]. Daytime hypoglycemia typically means the hypoglycemic event that occurs during the day. Postprandial hypoglycemia refers to the hypoglycemic event after the patient has eaten. It could also be referred to as reactive hypoglycemia, whereas nocturnal hypoglycemia means the hypoglycemic event occurs during the night when the patient is sleeping. Each type of hypoglycemia has its own associated risks. Patients run the risk of postprandial hypoglycemia when they misestimate the amount of carbohydrates (CHO) consumed in each meal. Varying insulin sensitivity is also a major factor in misanalysing the amount of bolus insulin needed and might lead to postprandial hypoglycemia [12]. Nocturnal hypoglycemia, on the other hand, is a much bigger problem than any other form of hypoglycemia. The reason is that nocturnal hypoglycemia occurs when the patient is sleeping and is virtually incapable of defending him-/herself against the glycemic event. The fact that over half of all the extreme hypoglycemic episodes occur during sleep add to the severity of this type of hypoglycemia.

Since hypoglycemia is a combination of various symptoms when blood glucose drops below 70 mg/dL and sometimes it is entirely asymptomatic, diagnosing it is very hard and it is near impossible for a human to predict its occurrence in advance. In the case of a hypoglycemic event, the initial treatment could be consuming 15 to 20 g of fast acting carbohydrates [2], and even though the consumption of glucose seems like the only solution to overcome an ailment that is caused by the deficiency of glucose, it takes 10–15 min for the human body to process glucose [4]. This means that the patient has already experienced mental and physical trauma before returning to a normal glycemic state. Moreover, clinical evidence and observational data show that the recommended glycated haemoglobin (HbA)targets are not met in the majority of T1D patients [13]. A more appropriate approach is to manage the blood glucose in such way that hypoglycemia is prevented.

There has been an immense surge in the use of technologies for diabetes management. Glucose monitoring systems have been one of the trending topics in biomedicine [14]. Multiple glucose monitoring devices are available these days that provide periodic or flash updates of the patient’s glucose levels. Some commercially available devices include Medtronic CGM, Abbott FreeStyle Libre, and Dexcom CGM systems [15,16]. These devices contain a continuous glucose monitoring (CGM) sensor along with a portable monitor that displays glucose levels and in some cases provides alarms of adverse glycemic events. The CGM sensors measure glucose dynamically and have a tiny filament inserted beneath the skin. These sensors remain in contact with the interstitial fluid with the help of an enzymatic electrode. Such electrodes use enzymes to cause reduction–oxidation reactions and then measure the amount of current or voltage produced by the movement of electrons, which is often concentration dependant [15]. The latest commercially available CGM sensors such as the FreeStyle Libre by Abbot give a BG value reading with a sampling time of 1 s and has a lifespan of 14 days after its first use, during which it does not need to be calibrated. This makes the process of testing BG less painful.

These monitoring devices are sometimes used in coordination with an insulin pump to form the sensor-augmented pump (SaP) therapy [17]. The SaP forms an important component of a closed-loop artificial pancreas (AP) system. Such systems have been worked upon for many years [18]. A closed-loop AP system has three main components, a CGM, an insulin pump, and control algorithm that controls the insulin dose. In other cases, the glucose monitoring systems, when used in coordination with an artificially intelligent decision-making module that gives suggestions about insulin and carbohydrate intake to the patients, form a decision support system (DSS). The DSS has proven to be an apt therapy for multiple daily injections (MDI) users, which is the most common method of insulin treatment for diabetic patients.

Machine learning (ML) has emerged as one of the major fields of artificial intelligence (AI) in recent times, and its impact on healthcare has been huge [19,20]. The concept of ML has its roots in computer science, statistics, and optimization. With a focus on enabling the computer to train itself without being explicitly programmed, ML gives a computer the power to predict outcomes up to a certain level of accuracy. In many medical scenarios knowledge of an adverse event beforehand could prevent an emergency and in many cases save lives. The quality of ML to predict the future makes it a great tool to anticipate such events [21]. Hypoglycemia, being one of such events, may also be anticipated using ML. The uncertainty associated with the occurrence of a hypoglycemic event looms on the horizon for T1Ds, making their lives ever so miserable. Biomedical engineers, therefore, want to come up with efficient predicting models in order to reduce the uncertainty and improve the life quality of diabetics. This is the reason that there has been an exponential increase in research work focused on ML techniques to predict adverse glycemic events in general and hypoglycemia in particular [22]. It is still too early to say that most such works are truly ready to be made commercially available for the public use; however, encouraging results have been seen in several of these works. It is known that ML techniques feed on large amounts of data in order for their prediction to be accurate. Moreover, the data need to be diverse and free of any corruption and irregularities [23]. To have such data for any biomedical application is a hard task because of the involvement of many such constraints that affect the quality of the data being acquired. Medical data are renowned for being complex and disordered. The limitations associated with sensors, noncompliance of patients to the study protocols, faults in the study protocols, and unwillingness of patients to undergo the study are some of the factors that affect the quality of data available for the training of ML algorithms. It is for this reason that biomedical data require a lot of pre-processing and filtration before being ready to be fitted with an ML model.

### The Aim of Hypoglycemia Prediction

Experts have tried to identify hypoglycemia based on different characteristics but most of the times, hypoglycemia is asymptomatic and is often unrecognized. This is one reason hypoglycemia can prove deadly. The absence of signs and prior indicators may cause the patients to act undesirably in the wake of a hypoglycemic event and consequently move themselves into disaster. Though the occurrence of hypoglycemia is hard to determine, it is often observed in patients who take insulin regularly [24]. Of the patients who take insulin, type 1 diabetics are three times more likely to experience hypoglycemia as compared to type 2 diabetics [25].

In many cases even when a hypoglycemic is recognized by the patient, it is often too late to prevent it. Hence, taking carbohydrates/glucose when a hypoglycemic event is taking place will not help the cause. It is therefore necessary to have a mechanism that could inform the patient in advance about the occurrence of a hypoglycemic event in the future. The aim of such a system should be to correctly forecast a hypoglycemic event in the future and then inform/warn the patient about it. A prediction system like this could be efficiently embedded in a decision support system (DSS). A DSS could then guide the patient about the steps and measures to be taken to prevent the predicted event from happening.

This review focuses on the performance and potential of several such works. The works that are reviewed here are explicitly focused on ML techniques for hypoglycemia prediction/detection in T1Ds. Table 1 and Table 2 shows the entire collection of manuscripts reviewed. It could be observed from these tables that the majority of the works done in the domain of hypoglycemia prediction/detection were published in 2019 and 2020. This is proof of a rising trend in the use of ML models for hypoglycemia prediction/detection. It is important to mention here that throughout this review, the ML frameworks are not discussed explicitly. No effort in establishing a ranking criterion has been made. The reason for this is that a large variety of ML frameworks are used in the literature and also, the factors defining the frameworks are diverse. Since no two studies used a common framework for ML modelling, comparing research works based on their frameworks was a hard task. Another reason of refraining from any sort of quantitative comparison was to keep the review as impartial as possible and let the readers establish an understanding of the work done in the field of ML-based prediction of hypoglycemia.

The methodology of the entire review process is discussed in the next section. Results obtained from the review are discussed in the section after that. A thorough analysis of the reviewed manuscripts is done in the results section based on a distinction between studies aimed at hypoglycemia detection and prediction, the data used to train the ML models, the type of ML models used, and the prediction/forecasting horizon. A discussion about the entire review is presented in the succeeding section followed by a conclusion of the presented work.

## 2. Materials and Methods

This paper looks at the broader horizon of the work done in the domain of ML for hypoglycemia prediction. The searched manuscripts were obtained by combining the results of multiple individual searches to form a pool of 900 manuscripts. PubMed and Google Scholar were used for the selection of manuscripts. PubMed was selected because it is the premier source of published research in biomedicine and life sciences available on the internet. Google scholar was selected for manuscript searching to enlarge the search area. English articles of the past five years were considered in this review. We excluded studies that involved type 2 diabetes or were review articles.

**Figure 1:**
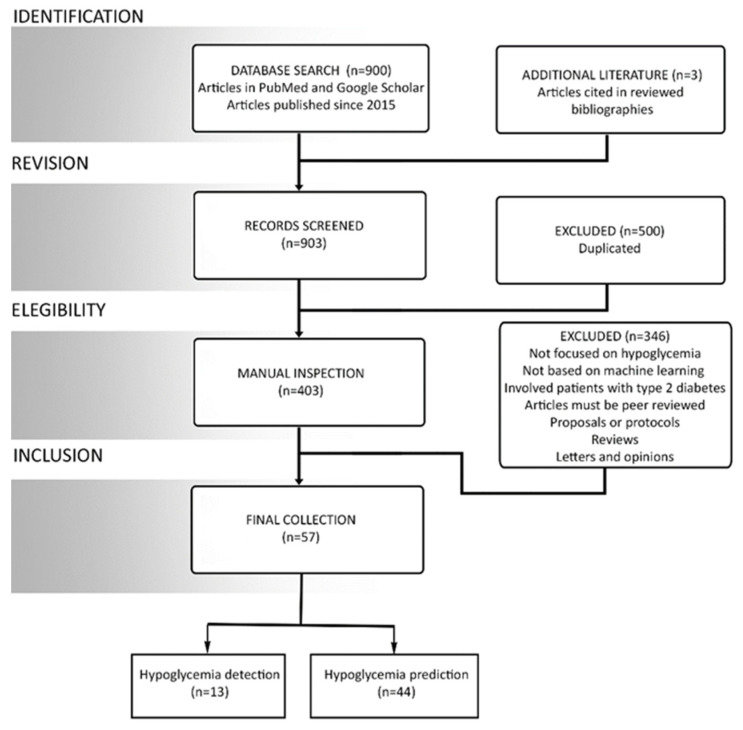
Overview of the review process and classification of literature.

Manuscripts were searched through the advanced searching options in PubMed. The search was carried out by first combining the keywords ‘machine learning’ and ‘hypoglycemia’ with the help of an ‘AND’ logical operator to search all the fields provided in PubMed advanced search option. This search yielded a total of 41 manuscripts. Later, keywords ‘artificial intelligence’ and ‘hypoglycemia’ were searched together for all the fields, which yielded a total of 47 manuscripts. ‘Machine learning’ was also searched together with ‘blood glucose prediction’, yielding a total of 119 manuscripts. The keywords ‘hypoglycemia’ and ‘machine learning’ were then searched together with a series of other keywords by using the same logical operator to obtain the following results: prediction (23), detection (10), hypoglycemic event (15), and adverse glycemic event (4). The keyword ‘hypoglycemia’ was then solely searched with other keywords using the logical operator yielding the following results: support vector machine (9), random forest (15), deep learning (8), ANN (6), supervised learning (21), and clustering (82). In google scholar, keywords ‘machine learning’, and ‘hypoglycemia’ were searched. This search was carried out to expand the pool of the total shortlisted manuscripts. A total of 500 manuscripts were searched using google scholar. All these individual searches were then combined together to form a grand pool of 900 manuscripts. Here, it is important to understand that the majority of the manuscript searched in both google scholar and PubMed were similar because both platforms provide distinct methods of article searching. A thorough review of the selected manuscript pool was performed. Moreover, the bibliographies of the selected manuscripts were looked into for a detailed analysis of the manuscripts cited in these works. The shortlisted manuscripts were then scrutinized to obtained the final collection of 57 papers by using the methodology given in Figure 1.

## 3. Results

Results were obtained from the 57 shortlisted papers after a comprehensive analysis of the attributes that were found to be most impactful in deciding the quality of the work. The details of all the reviewed manuscripts are given in Table 1 and Table 2. The results section is based on the following categories:

- The prediction and detection of hypoglycemia
- Type of data
- ML models
- Prediction horizon (PH)

### 3.1. The Prediction and Detection of Hypoglycemia

The first major classification of the manuscripts reviewed was done on the basis of where in time the ML models look for the occurrence of a hypoglycemic event. Hypoglycemia prediction essentially means forecasting the future hypoglycemic events. On the other hand, the detection of hypoglycemia only means detecting whether a hypoglycemic event has occurred at the present time or not. Many ML-based systems are just detection models. They do not look into the future to forecast the occurrence of an event. Even though this review primarily focuses on discussing the prediction models, the importance of detection models cannot be undermined. Automatic real-time detection of hypoglycemia may be crucial in many scenarios. In this section, works whose aim was to recognize or estimate the occurrence of a hypoglycemic event in the present have been identified. The purpose of doing so was to narrow down the review towards the works that were only focused on hypoglycemia prediction in the sections ahead. It is important to take into consideration that in order to have an ML algorithm that forecasts future events we must have time series data. In the context of this review, this is equivalent to saying that in order to predict the occurrence of a hypoglycemic event at a specific time in the future, the data used to train the ML models need to contain the BG, insulin, CHO or some other form of time series data.

From this, it can be deduced that works that do not use time series data do not try to predict the occurrence of hypoglycemia in the future but most often than not try to detect hypoglycemia in the present. The details of such works are given in Table 1. Physiological parameters of an electrocardiogram (ECG) were used to detect hypoglycemia by Ling et al. [42], Ranvier et al. [34], and San et al. [35]. Multiple systems used text and language processing for the detection of hypoglycemia. For instance, hypoglycemia was detected from electronic health records (EHRs) in the investigations proposed by Jin et al. [29], Ruan et al. [31], and Jin Li et al. [34]. Chen et al. [30] employed patient secure messages for automatic detection of hypoglycemia while Zhou et al. [26] aimed at detecting hypoglycemia by processing the text of clinical notes of patients. Temperature, near infra-red, and bio impedance sensors were employed in their system for the detection of BG trends during the occurrence of a hypoglycemic event by Tronstad et al. [28]. Marling et al. [32] used heart rate (HR), temperature, and galvanic skin response (GSR), and Juhl et al. [37] utilized electroencephalogram (EEG) data to perform hypoglycemia detection.

### 3.2. Type of Data: What Are the Current Models Trained on?

ML engineering primarily involves fitting ML models to a large amount of data in order to locate patterns and classify them into different label groups. For an ML model to work efficiently, a large quantity of good relevant data is required, which means that the data used to train ML models should be accurate, complete, and valid. However, in biomedical applications, the availability of good data for ML designers is rare, the reason being different natural and technical constraints involved in the process of data collection.

**Figure 2:**
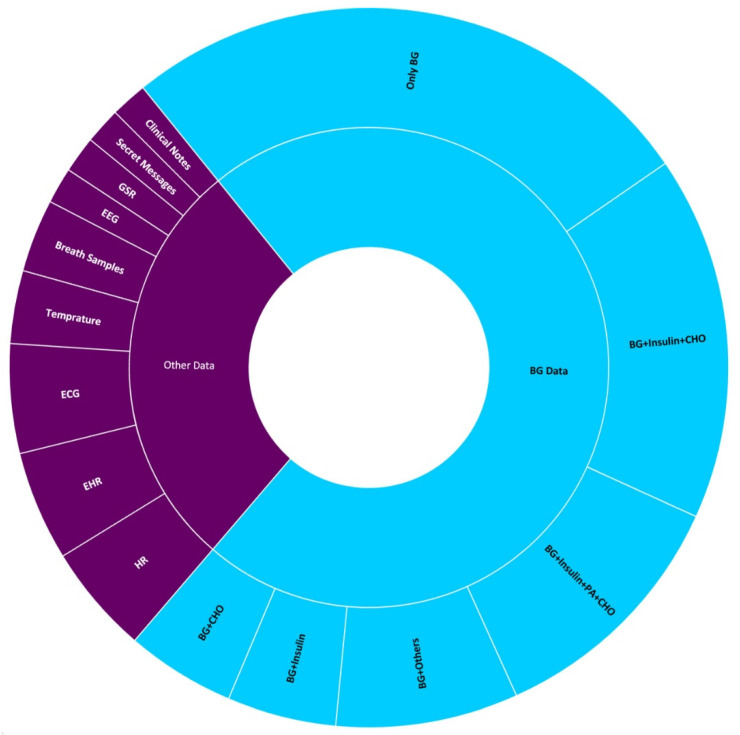
Different types of data used to train ML models.

ML models for hypoglycemia prediction/detection may be trained on several types of data. The manuscripts we have reviewed used 12 different types of data to train ML models. These data include BG, insulin, carbohydrates (CHO), ECG, EHRs, HR, breath samples, temperature, clinical notes, secret messages, GSR, and EEG. Figure 2 shows the distribution of the number of manuscripts for each type of data. It must be kept in mind that by data we mean the acquired data in their original form and not the extracted features.

#### 3.2.1. Blood Glucose (BG) Data

As one might think, the most relevant data while predicting events based on BG levels would be the BG values itself. Based on the profile of an individual’s BG levels an ML system could be trained to predict the future BG values. This approach was used by the majority of the works that have been reviewed. Approximately 77% of the total manuscripts reviewed use BG data to train ML models for hypoglycemia prediction. It is, however, important to mention that not all of these models were trained on actual clinical data. Actual clinical data come from clinical trials. These trials are overseen by a clinical trial protocol that describes the terms and conditions under which the study is ought to be conducted. Some of these works use BG data from diabetes patient simulators. Diabetes simulators are platforms that are used to emulate certain physiological characteristics of a diabetic patient and allow the user to perform experiments by controlling different parameters related to insulin dosing strategies for diabetes patients. Diabetes simulators are often preferred in pre-clinical trials to evaluate the performance of new diabetes management systems/strategies. Some of the famous diabetes simulators include the UVA/PADOVA simulator and Hovorka model, etc. Of the works based on BG data reported in this review, 13.63% use simulated BG data from different diabetes patient simulators, while 68.18% use actual clinical data, whereas 18% of the manuscripts use both real and simulated patient data.

Moreover, of the works that use BG for hypoglycemia detection, some make use of sole BG data while others use BG plus a combination of different types of data such as insulin, CHO, and PA data, etc. The distribution of different BG data combinations may be observed in Figure 2. About 36.36% of the manuscripts used BG data alone for hypoglycemia prediction; 6.82% of manuscripts predicted hypoglycemia by using BG combined with insulin data, and an equal amount of research work used BG combined with CHO. A total of 22.73% works used BG combined with insulin, CHO, and PA data. A total of 15.91% studies used BG, insulin, CHO, and PA data in conjunction while 11.36% studies made use of BG in combination with other sources of data, e.g., HR and ECG, etc.

#### 3.2.2. Only BG

The details of the works that trained ML models only on BG data are given in Table 2. Most of these models are time series forecasting models and involve BG data that have certain timestamps associated with the actual BG values. Nocturnal hypoglycemia prediction was targeted in studies such as Kriukova et al. [44], Vu et al. [47], Sampath et al. [69], and Tkachenko et al. [49]. Seo et al. [43] proposed the prediction of postprandial hypoglycemia by training ML models with BG data while Jung et al. [54] predicted day-time hypoglycemia using similar data. Quan et al. [50], Dong et al. [62], and Mhaskar et al. [63] used neural networks trained on BG data for hypoglycemia prediction. On the other hand, Rodriguez et al. [57] used three different ML models trained on BG data from 25 patients. A KRR-based system was presented by Marcus et al. [58] while Seo et al. [46] proposed another model to predict hypoglycemia that used BG values.

#### 3.2.3. BG Combined with Other Types of Data

From Figure 2 it is evident that of the manuscripts which use BG data for training, a portion use the combination of BG and insulin data. Jensen et al. [39] and Mosquera-Lopez et al. [48] proposed models that predicted nocturnal hypoglycemia from BG and insulin together. These systems showed moderate performance in terms of sensitivity and specificity. A recurrent neural network (RNN) was trained by Mosquera et al. [81] using BG and insulin data for adverse glycemic event prediction. This system was reported to be more than 90% accurate in predicting hypoglycemic events.

Some studies trained ML models on BG along with CHO values. The quantity of such studies is very low since the CHO data are often very inconsistent and not a lot of ML designers like to work with them. CHO, however, is an important feature to consider in insulin prediction models. Insulin bolus calculation was performed by Zhu et al. [65] and Giulia Noaro et al. [55]. Both of these works employed BG and CHO data. Zhu et al. [73] presented a system based on DRL. This study displayed an improved control of single hormone and dual hormone insulin delivery.

Other works such as Dave et al. [52], Shifrin et al. [56], and Cappon et al. [66] used BG along with insulin and CHO for prediction purposes. Aiello et al. [67] and Oviedo et al. [53] both aimed at postprandial hypoglycemia prediction by utilizing BG data combined with insulin and CHO data. Noaro et al. [72] proposed an insulin bolus calculator while Vehi et al. [59] proposed a hypoglycemia prediction and prevention system that employed BG, insulin, and CHO data for ML model training. A DSS that provides weekly insulin dosage recommendations for type1 diabetics was proposed by Tyler et al. [61].

There are certain works that along with BG, insulin and CHO made use of additional data e.g., physical activity (PA) data and HR etc. Nocturnal hypoglycemia was predicted from BG, insulin, CHO and PA data by Calhoun et al. [45], Bertachi et al. [51], Bertachi et al. [41] and Güemes et al. [60]. Glucose value forecasting is performed by Li et al. [78], Mayo et al. [71], Zhu et al. [64] and Daskalaki et al. [70] through the utilization of such a combination of data.

Certain works have also used BG data in combination with other data such as breath samples and camera samples, etc. Reddy et al. [40] predicted hypoglycemia at the start of an aerobic exercise. Hypoglycemia was predicted from breath samples using ML techniques by Siegel et al. [27]. Vahedi et al. [33] predicted BG levels from BG and PA while BG levels were estimated from PPG signals using the mobile phone camera of a patient and the BG data in a study proposed by Zhang et al. [38].

#### 3.2.4. Other Types of Data

We are aware that the beauty of ML lies in formalizing non-linear relationships between different data(s) and outcomes. Researchers have hence tried to predict hypoglycemia by training ML models using multiple types of other data. Of the works that we have reviewed, 22.81% are based on data other than BG as shown in Figure 2. These data include the EHR, ECG, GSR, EEG, clinical notes, secret messages, breath samples, and body temperature. The individual percentages of works based on these data are as follows: EHR 5%, ECG 5%, HR 5%, breath samples 3.5%, body temperature 3.5%, clinical notes 2%, secret messages 2%, GSR 2%, EEG, 2%.

### 3.3. Machine Learning Models

**Figure 3:**
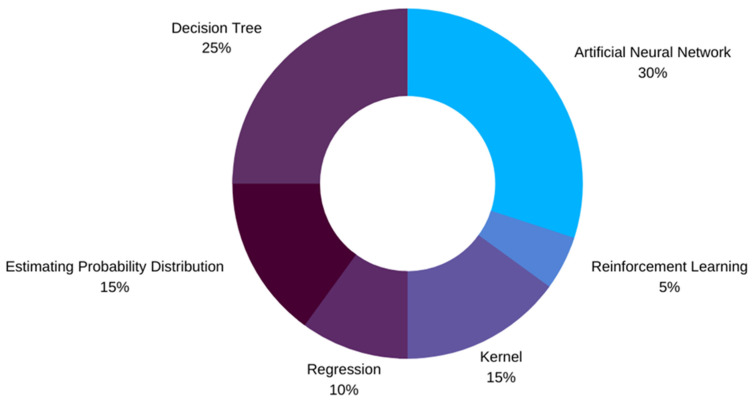
ML models used by studies, grouped into families based on similarity.

ML designers have a variety of ML algorithms at their disposal while implementing new designs. The choice of an ML algorithm is guided by multiple factors, i.e., the type of data used to train the model, the number of features, and most importantly, the quantity of data available [82]. The literature reviewed here shows that a total of 34 unique ML algorithms have been used as can be observed in Table 1 and Table 2. These algorithms have been categorized into six major families of ML algorithms as shown in Figure 3. The most common of these families is the ANNs followed by the DTs, kernels, and others. If we talk about the most famous individual ML models, RF has been the choice of designers followed by SVM.

The majority of the studies have utilized the self-learning capabilities of ANN. These studies have employed multiple variants of ANN such as RNN, DL, CNN, MLPs, etc. ANNs were used by Bertachi et al. [41], Vahedi et al. [33], Zhu et al. [64], Mosquera-Lopez et al. [81], San et al. [35], Jin et al. [36], Mhaskar et al. [63], Li et al. [74], Li et al. [78], Bertachi et al. [51], Güemes et al. [60], Oviedo et al. [53], Vehi et al. [59], Quan et al. [50], and Amar et al. [75]. Unlike other ML models, ANNs extract their own features from the inputs based on their hidden parameters. ANNs were used together with a reinforcement learning algorithm in studies presented by Zhu, Li, Kuang, et al. [65], and Zhu, Li, Herrero, et al. [73].

DTs are predictive models that predict the outcome for a set of input features after testing the features through several tree branches. DTs too have multiple variants that were utilized in the studies cited in this review. The most famous variant of DT is RF. Because of characteristics such as robustness to noise, handling of missing values and robustness to outliers, RF has been chosen by many ML designers for this application. RF creates a large number of decision trees and then outputs the mode of all the decision trees. This approach fixes the over-fitting problem of decision trees. Seo et al. [43], Güemes et al. [60], Vahedi et al. [33], G Noaro et al. [72], Vu et al. [47], Reddy et al. [40], Chen et al. [30], Dave et al. [52], Calhoun et al. [45], Amar et al. [75], Hidalgo et al. [77], and Rodriguez et al. [79] have all used RF for predicting/detecting hypoglycemia. Ruan et al. [31] and Cappon et al. [66] used the XGboost algorithm. XGboost is the gradient-boosted variant of DT and is aimed at enhancing the performance of decisions trees. Common DTs were employed by Ranvier et al. [34] and Reddy et al. [40].

Kernel-based SVM is the second most common choice of ML algorithms for designers working towards the goal of hypoglycemia detection/prediction. This is an indicator of the fact that SVM works well for such problems where the data-sets are relatively small. SVM is a binary linear classifier that maps feature points in space, creating different categories [83]. These categories are separated by a gap as wide as possible. When a test point is brought to the model, SVM maps it to one of the various categories and then assigns it a label. Marling et al. [32], Mosquera-Lopez et al. [48], Seo et al. [43], Güemes et al. [60], Oviedo et al. [68], Vehi et al. [59], Chen et al. [30], Bertachi et al. [41], and Rodriguez et al. [79] have all used SVM.

Regression techniques in ML predict the result of a continuous output variable. In the case of LR, however, the output is often a discrete label. The various types of regression used by studies in this review are LR, GE, and MLR. Studies proposed by Chen et al. [30], Dave et al. [52], and Seo et al. [43] use LR. LR fits a logistic function to data and outputs the probability of one or more classes. KNN is another ML approach that was used in several studies, such as Tyler et al. [61], Zhang et al. [36], Aiello et al. [67], Seo et al. [43], and Hidalgo et al. [77]. KNN looks for the closest examples in the feature space and then assigns them a label. Jensen et al. [39] and Siegel et al. [27] used LDA for prediction purposes. LDA is often used for the purpose of dimensionality reduction in classification problems.

It is important to consider that ML models can be evaluated with a range of different performance metrics. It is, therefore, impossible to present a quantitative performance comparison of the reviewed literature since the performance metrics differ for different works. Sensitivity and specificity have been the researchers most favorite performance metric with 47% of the studies using it, followed by root mean square error (RMSE), in 21% of the research works reviewed. Accuracy was used as a performance metric in a total of 13% of the manuscripts, similar to the area under the ROC curve (AUC). The mean absolute percentage error and blood glucose risk index were each used to evaluate 3.3% of the total manuscripts.

### 3.4. Prediction Horizon: How Far Are the Current Systems Forecast in the Future?

**Figure 4:**
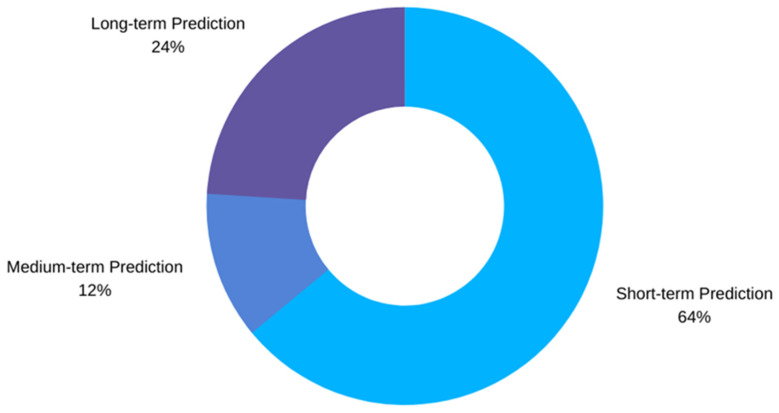
Different types of PH categories used in the studies.

In ML analysis of time series data, PH or forecasting horizon is the amount of time the user has before the occurrence of a predicted event. In biomedical applications, ideally, the PH should be large enough to give the patient apt time to take preventive measures and prevent an adverse event from happening. In the case of hypoglycemia, if the ML algorithm predicts the occurrence of a possible hypoglycemic event 30 min from the time of prediction, the user only has 30 min to take necessary actions in order to prevent the predicted hypoglycemic event from happening. In the mentioned case, whether the PH of 30 min is enough time or not is a debate that is dependent on various factors such as the severity of the hypoglycemic event, effectiveness of medications, and the amount of CHO consumption. The PH defined in a particular approach has two important effects on the achieved predictions: the time a patient has to respond and the error associated with estimations increase together. Therefore, it is extremely important to find a balance between the error we are willing to take and the requirements of our approach. There are a total of 14 different PHs reported in the literature reviewed with the PH of 30 min being the most common, followed by PH values of 6 h, 60 min, and 15 min. The PH values are categorized based on short-term, medium-term, and long-term predictions. The PH categories based on their frequency of usage are provided in Figure 4. The details about all the PHs reported in the literature are given in Table 3.

## 4. Discussion

After an organized analysis of high quality research work in PubMed and Google Scholar, we pinned down manuscripts with an aim of providing a thorough overview of the work done in the field of ML for predicting hypoglycemia. The review demonstrates that the use of ML models for the prediction of hypoglycemia has increased considerably over the last five years. It was observed that not all the manuscripts reviewed focused on predicting hypoglycemia. Some of the works only focused on the detection of the glycemic event. Technically, detection of an event could be referred to as description. It is important to understand that hypoglycemia prediction is BG level prediction in essence. Hypoglycemia is but a condition labelled on the predicted BG value graph. That is precisely the reason why some of the works that we have reviewed are BG level prediction models and do not talk about hypoglycemia prediction explicitly. These works, however, do provide a framework for the prediction of hypoglycemia.

A correlation between the type of data used for training the ML model and the nature of output (description/prediction) suggests that ML time series prediction is only possible with time series data such as BG, insulin, and CHO, while detection could be performed using other types of data such as breath samples, EEG, PPG, etc. Data acquisition in biomedical applications suffers from multiple constraints such as hardware limitations, restricted clinical environments, failure of patients to comply with study protocols, and obstacles in the way of large biomedical data collection. These barriers compel ML designers to work with the available imperfect data and look for solutions. The issue of imperfect data may be tackled through different strategies. The missing values problem is often addressed by using some kind of interpolation or imputation method. Prediction of missing values based on other values is also a technique that has been used to address this problem. Different types of regression or classification models can be used to predict missing values. Deep learning-based imputation is often preferred because of its accuracy. In various studies a certain range of missing values is selected to perform interpolation. Any gap in the data that exceeds that limit of missing values is then termed missing data and no interpolation is done. Frameworks based on conditional probability such as the theory of belief function, evidence theory or linear belief functions can be used to address the problem of incomplete data or missing data.

In this review, the assessment of data used in training exhibits a slanted picture with BG data dominating most of the reviewed studies. It has been observed that studies that use data other than BG are almost all targeted at detecting hypoglycemia. There are claims by some works of predicting hypoglycemia while using data other than BG, but a thorough inspection revealed that the targeted PH was either too small to be classified as real prediction or it does not exist at all. It is also worth mentioning here that many works reported an issue with the acquired data in terms of size or completeness. The need for data that are both large in size and good in quality is ultimate. It is known that ML models map complex nonlinear relationships in physiological data to perform prediction or description. To perform this nonlinear mapping, the required data have to be complete and relevant. In particular, BG value detection and prediction feed on data obtained from various types of sensors, i.e., CGM and HR sensors, etc. Two of the most common issues with CGM sensors is the sensor delay and sensor malfunctions. Sensor delay in CGM is the inherited 10-min discrepancies, while sensor malfunctions are those periods in which no BG value is recorded. The quality of these sensors is one area that needs to be improved in future.

An in-depth analysis of ML models presented a broader picture of the preferred techniques for the purpose of hypoglycemia prediction. It is understood that the quality and quantity of data affect the choice of the ML model. Since the data in this case suffer from various issues, the choice of ML model should be made such that it makes up for the deficiencies in the data. Models such as SVM are preferred because of their ability to handle a relatively small amount of data with greater efficiency. ML models are also chosen based on the level of complexity. Simpler models such as RF and KNN are preferred because they give good results most of the time and are easier to implement. Moreover, the reason that the majority of the works use RF and SVM is that these algorithms provide a higher level of versatility in terms of the type of problem they are used for. On the other hand, ANNs are data hungry and in the case of hypoglycemia prediction it is observed that efforts are made to train the network on large datasets. DL is an area that has not been used extensively for hypoglycemia prediction and can be explored more in future. Gradient-boosted tree algorithms such as XGB are nowadays preferred by designers for time series analysis if the time series problem is a supervised learning problem. The use of multiple gradient-boosted algorithms can be observed in the review. The quest for improved results is analogous to training different ML models. Designers, therefore, have trained various other models in the work reviewed.

It is understood from the review that, for a hypoglycemia DSS, the most important trait to have is to warn the patient about a hypoglycemic episode well before it happens. Early detection helps the patient cope with the hypoglycemic event in a better way. PH is a term closely associated with time series data forecasting. How far does an ML-based prediction model see into the future is a thing to consider before passing any verdict about the quality of the model. The length of a PH correlates with the amount of data an ML model is trained on. Ranging from a PH length of 15 min to 1 week, designers have tried to predict hypoglycemia in different future time frames. The race for a longer PH is always on but the desirable choice of PH mostly depends on the nature and type of application.

## 5. Conclusions

ML for the prediction of hypoglycemia has been trending topic among biomedical data engineers. Our review demonstrates the potential impact such predictive models could have in the field of diabetes healthcare. A highly efficient hypoglycemia predictor may prove life-changing for T1Ds. The timely prediction of a hypoglycemic episode can immensely improve the life quality of T1D patients and on top of that, save their lives. There has been a stark increase in the amount of research work done in the area of hypoglycemia prediction using ML. This is evident from the increasing number of studies published in this domain during the past five years as depicted by this review. Though ML models appear to be the right choice for figuring out the nonlinear relationships between different types of physiological data and the occurrence of hypoglycemia, there is still room for improvement. This review gives an insight into the challenges faced by the designers while dealing with imperfect data for hypoglycemia prediction and detection. The results obtained from this review provide an overview of the go-to ML models for researchers while predicting/detecting hypoglycemia. Discussion of the PH portrays a picture regarding how far the current systems predict hypoglycemia in the future. It is concluded that ML for hypoglycemia prediction holds considerable potential. Research in this domain must continue and more directions should be explored. Researchers are advised to further explore this domain by training different ML models on various types of sensor data. In the context of hypoglycemia prediction, it is paramount to come up with new strategies to train ML models with more data. ML engineers could use a two-phase training approach by first training the ML models with a huge amount of data (populational models, similar patients, virtual patients, generated data, etc.) and then training the ML models with more specific data such as cohort data, real time data, etc. Creativity in feature engineering and techniques for the acquisition of healthy datasets are areas that need to be worked on for the realization of accurate ML-based hypoglycemia predictors. The incorporation of such ML models in DSS should be ensured and made available for the benefit of patients.

## Figures and Tables

**Table 1:** Summary of reviewed manuscripts addressing hypoglycemia detection: ML model used to perform the detection, type of data (ToD) used to train the ML model, the size of the cohort, recording duration (RD), age of population (AoP), gender of the participants in study, treatment method (TM).

Ref	Year	ML Model	ToD	Cohort	RD	AoP	Gender	TM
[26]	2019	cTAKES	clinical notes	395 and 460 notes	-	-	-	-
[27]	2017	LDA ^a^	BG, breath samples	56	1 bag each	CH ^n^, ADO ^p^, ADU ^q^	F ^r^: 55.36%, M ^s^: 44.64%	-
[28]	2018	PLSR ^b^, ANN ^d^	temp, IR ^c^, Z	20	2 days	ADU	-	-
[29]	2018	CNN ^e^	EHR	500 records	95,246 sentences	-	-	-
[30]	2019	LSVM ^f^, LR ^g^, RF ^h^	Secure messages	3000 messages	-	-	-	-
[31]	2020	XGBoost	EHR	17,658	4 years	ADU	F: 47% M: 53%	MDI
[32]	2016	SVM ^i^	HR, temp, GSR	1	2 months	ADU	M: 100%	IP ^u^
[33]	2018	RF, MLP ^t^	BG, PA	93	4 months	CH, ADO, ADU	F: 46.2% M:53.7%	IP
[34]	2016	DT ^j^	ECG, breath data, accelerometer	5	260 h	-	-	IP
[35]	2016	DL ^k^	ECG	15	10 h	CH	-	MDI
[36]	2019	DL	EHR	500 records	-	-	-	-
[37]	2015	-	EEG	15	-	CH, ADO, ADU	-	-
[38]	2019	KNN ^l^	camera, BG	14	850 samples/subjects	ADU	-	-

^a^ LDA: Linear Discriminant Analysis; ^b^ PLSR: Partial Least Square Regression; ^c^ IR: Infra-Red; ^d^ ANN: Artificial Neural Network; ^e^ CNN: Convolutional Neural Network; ^f^ LSVM: Linear Support Vector Machine; ^g^ LR: Logistic Regression; ^h^ RF: Random Forest; ^i^ SVM: Support Vector Machine; ^j^ DT: Decision Tree; ^k^ DL: Deep Learning; ^l^ KNN: K-Nearest Neighbor; ^n^ CH: Children; ^p^ ADO: Adolescents; ^q^ ADU: Adults; ^r^ F: Female; ^s^ M: Male; ^t^ MLP: Multilayer Perceptron.

**Table 2:** Summary of reviewed manuscripts addressing hypoglycemia prediction: ML model used to perform the detection, prediction horizon (PH) in minutes, the size of the cohort, recording duration (RD), type of data (ToD) used to train the ML model, treatment method (TM), age of population (AoP), gender of the participants in study.

Ref	Year	ML Model	PH (min)	Cohort					
				Size	RD	ToD	TM	AoP	Gender
[39]	2019	LDA	35	463	4721 nights	BG, Insulin	IP	ADU	F: 58% M: 42%
[40]	2019	DT, RF	30	55	244 exercise sessions	HR, BG	IP	ADU	F: 60% M: 40%
[41]	2020	MLP, SVM	360	10	12 weeks	HR, BG, CHO	MDI	ADU	F:80% M:20%
[42]	2016	Extreme ML NN	360	16	4.09 days	ECG	-	CH	-
[43]	2019	RF, SVM, KNN, LR	30	104	113 days	BG	MDI	ADU	F: 60% M: 40%
[44]	2017	k-mean clustering	540	34	10 days	BG	-	ADU	-
[45]	2020	RMRF ^a^	-	127	2525 nights	BG, PA, Insulin, CHO	-	-	-
[46]	2019	Ensemble of commonly used ML models	30	104	Between 2014 and 2015	1 (BG)	-	-	-
[47]	2020	RF	360	9800	1 mil nights	BG	IP	ADU	F: 51% M: 49%
[48]	2020	SVR ^b^	360	124	22,804 nights	BG, Insulin	IP	ADO, ADU	F: 60% M: 40%
[49]	2016	stochastic models	60, 240, 360	34	150 days	BG	MDI	CH, ADU	-
				179	476 days				
[50]	2019	ANN	30	N/A	1 Week	BG	-	-	-
[51]	2018	ANN	30, 60	6	8 weeks	Insulin, BG, PA, CHO	IP	ADU	-
[52]	2020	LR, RF	0–15, 15–30, 30–45, 45–60	112	90 days	BG, Insulin, CHO	IP	ADO, ADU	F: 39.2% M: 60.7%
[53]	2019	ANN, SVM, AB ^c^, GNB ^d^	240	10 10	Several months	BG, PA, Insulin, CHO	MDI	ADU	-
[54]	2017	CART	15	33	72 to 96 h	BG	-	-	-
[55]	2020	MLR, LASSO	420	100	162,000 traces	BG, CHO	-	ADU	-
				218	2 months				
[56]	2020	MDP ^e^	210	NIDDK repository	6 Treatment points	BG, Insulin, CHO	-	ADU	-
[57]	2019	ARIMA, RF, SVM	15	25	14 days	BG	IP	ADU	F: 44% M: 56%
[58]	2020	KKR ^f^	30	11	7–50 days	BG	IP	ADU	-
[59]	2019	GE ^g^, SVM, ANN	60	100	14 days	BG, Insulin, CHO, PA	IP	ADU	-
			240	10	6 weeks				
			360	6	8 weeks				
[60]	2019	RF, SVM, ANN	120	6	8 weeks	Insulin, BG, PA, CHO	IP	ADU	-
[61]	2020	KNN	10,080	70	15 weeks	BG, insulin, CHO	MDI	-	-
[62]	2019	GRU ^h^	45	40	4 days	BG	-	-	-
[63]	2017	DL	30	25	N/A	BG	-	CH, ADO	-
[64]	2020	RNN ^i^	30	10	360 days	Insulin, BG, PA, CHO	IP	-	-
				6	8 weeks				
[36]	2019	RNN	30	124	27,466 days	BG, Insulin	IP	ADU	-
[65]	2020	DRL ^j^	The meal duration	10 10	6 months	BG, CHO	MDI	ADO, ADU	-
[66]	2019	XGBT ^q^	The meal duration	100	2 months	BG, Insulin, CHO	-	ADU	-
[67]	2019	KNN	The meal duration	100	4 days	BG, Insulin, CHO	-	ADU	-
[68]	2019	SVM	The meal duration	10		BG, Insulin, CHO	IP	ADU	F:20%, M: 80%
[69]	2016	Combination of NH predictors	360	34	150 days	BG	MDI	CH, ADU	-
				179	476 days				
[70]	2016	ACL ^k^	1440	28 100		BG, PA, Insulin, CHO	-	CH, ADO, ADU	-
[71]	2019	10 Different ML Methods	30	6	8 weeks	Insulin, BG, PA	IP	ADU	-
[72]	2020	RF, GBT	The meal duration	100	162 meal conditions	BG, Insulin, CHO	-	-	-
[73]	2019	DRL	N/A	10 10	30 days	CGM, CHO	-	ADO, ADU	-
[74]	2019	DL	30, 60	10	6 months	BG, Insulin, CHO	IP	ADO, ADU	-
				6	8 weeks				
				10	180 days				
[75]	2020	ARM ^r^, RF, LGBM ^l^, FCNNs ^m^,GCNN ^n^	30, 60	141	9083 days	BG	-	-	-
				30	30 days				
[76]	2018	ANN	30	12	1 year	BG	IP	ADU	F: 50%, M: 50%
[77]	2017	GP ^o^, RF, KNN, GE	30	10	N/A	BG, insulin, CHO	-	-	-
[78]	2020	DL	30, 60	10 10	6 months	BG, Insulin, CHO, PA		ADU	-
[79]	2019	RF, SVM	15	25	14 days	BG	MDI	ADU	F: 44%, M: 56%
[80]	2017	LSTM ^p^	30, 60, 90	106	7 days	BG	Both	-	-

^a^ RMRF: Repeated Measures Random Forest; ^b^ SVR: Support Vector Regression; ^c^ AB: Adaboost; ^d^ GNB: Gaussian Naïve Bayes; ^e^ MDP: Markov Decision Process; ^f^ KKR: Kernel Ridge Regression; ^g^ GE: Grammatical Evolution; ^h^ GRU: Gradient Recurrent Unit; ^i^ RNN: Recurrent Neural Network; ^q^ XGBT: Extreme Gradient Boosted Tree; ^k^ ACL: Actor Critic Learning; ^l^ LGBM: Light Gradient Boosting; ^m^ FCNN: Fully-convolutional Neural Networks; ^n^ GCNN: Gradually Connected Neural Networks; ^o^ GP: Genetic Programming; ^p^ LSTM: Long Short-term Memory; ^q^ ARM: Autoregressive Model.

**Table 3:** Prediction horizons used by different works.

Prediction Type	Manuscript	Prediction Horizon (PH)
**Shor-Term Prediction**	[52,54,57,79]	15 min
	[40,43,46,50,51,52,58,63,64,71,74,75,76,77,78,80,81]	30 min
	[39]	35 min
	[52,62]	45 min
	[49,51,52,59,74,75,78,80]	60 min
**Medium-Term Prediction**	[80]	90 min
	[60]	120 min
	[56]	210 min
	[49,53,68]	240 min
**Long-Term Prediction**	[41,42,47,48,49,69]	360 min
	[55]	420 min
	[44]	540 min
	[61]	1 Week
	[66,67,72]	Meal Duration

## Data Availability

No new data were created or analyzed in this study. Data sharing is not applicable to this article.
